# Extrahepatic metastases of hepatocellular carcinoma on ^18^F FDG PET CT

**DOI:** 10.1186/s43046-021-00086-0

**Published:** 2021-11-26

**Authors:** Manjit Sarma, Subramanyam Padma, Parvathy Pavithran, Vijay Harish Somasundaram, Palanisamy Shanmuga Sundaram

**Affiliations:** Department of Nuclear Medicine & Molecular Imaging, Amrita Institute of Medical Sciences, (Amrita Vishwa Vidyapeetham), Cochin, Kerala 682041 India

**Keywords:** Hepatocellular carcinoma, Extrahepatic metastasis, FDG PET CT

## Abstract

**Background:**

To determine locations, relative frequencies, imaging features, and pattern of distribution of extrahepatic metastasis from hepatocellular carcinoma (HCC) on 2-deoxy-2-[fluorine-18]fluoro-D-glucose (^18^F-FDG) PET CT.

**Methods:**

FDG PET CT scans of 224 consecutive patients of HCC acquired between 2010 and 2018 were reviewed. Fifty-six patients detected with extrahepatic metastasis on FDG PET CT were retrospectively analyzed. Findings were correlated with prior/follow-up imaging studies, clinical findings, FNAC, or biopsy findings whenever available. Descriptive analysis of location, relative frequencies, imaging features, and pattern of distribution of extrahepatic metastasis was done.

**Results:**

Commonest were metastatic pulmonary nodules (55.3% patients), most of them being well-defined solid lesions (53.5%) with bilateral involvement in 44.6% patients and lower lobes of lungs along with other lobes being more frequently involved (41.0% patients). While in 7.14% patients lung nodules were FDG avid, 23.2% patients had both FDG avid and non-avid pulmonary nodules. Second most common were regional metastatic lymph nodes in 44.65% of patients seen at aortocaval (25%), paraaortic (23.21%), portocaval (21.4%), and left gastric nodal (17.8% of patients) stations. Twenty-five percent of patients had FDG avid lymph nodes and 5.36% patients had both FDG avid and FDG non-avid lymph nodes. Distant metastatic lymph nodes were third most common in 39.2% of patients seen at paratracheal (2.5%), juxtaphrenic (8.9%), and mesenteric lymphnodal (7.1%) stations. Twenty-five percent of patients had FDG avid lymph nodes while 5.36% patients had both FDG avid and FDG non-avid lymph nodes. Skeletal involvement was seen in 32.1% of patients. Commonest sites are vertebrae (16.7%), pelvis (14.2%), and ribs (10.7% patients). Six out of 7 patients had unilateral adrenal gland involvement. Bilateral adrenal gland involvement was seen in 1 patient. FDG non-avid peritoneal/omental metastases was seen in 2 patients. Brain, spleen, and muscle metastatic lesions were seen in 1 patient each out of 56 patients (1.79%).

**Conclusions:**

Lungs, regional and distant lymph nodes and skeleton are the most frequently involved sites of extrahepatic metastatic hepatocellular carcinoma. Adrenal glands, muscles, brain and peritoneum are also involved but to a lesser extent.

## Background

Extrahepatic metastases are known to occur in 14.0 to 36.7% of patients with HCC [1]. Detection of extrahepatic metastases is critical in deciding optimal treatment [2] and extrahepatic metastases is also known to be an independent predictor of poor survival [3]. ^18^F FDG whole-body PET CT is being increasingly used for various indications in many malignancies but its use in HCC remains controversial because of concerns about the relatively low sensitivity, especially for detecting primary well-differentiated HCC [4]. Since there are reports of FDG PET CT being able to detect 92.9% of extrahepaticmetastases larger than 1 cm in largest dimension [5], its use as a staging tool would especially be relevant for developing countries, which bear the major brunt of HCC incidence (≥ 80%), for optimal utilization of resources [6]. Locoregional therapies (like resection, ablation, or liver transplantation) may not always be suitable and would in fact be adding to the financial burden of patients from these countries. As effective detection of extrahepatic metastases has huge management implications [7], knowledge of the location, relative frequencies, patterns of distribution, and radiologic appearance of the extrahepatic spread of HCC would be important in order to assure the choice of the most appropriate therapy and therefore to assure patients the best chance for longer survival [8]. In this study, we attempt to demonstrate the clinical relevance of ^18^F FDG whole-body PET CT in initial evaluation of HCC patients in a developing nation.

## Methods

### Patients

FDG PET CT scans of 224 consecutive patients of HCC acquired between September 2010 and May 2018 were retrospectively reviewed. Out of them, FDG PET CT scans of 56 patients who were detected to have extrahepatic metastases from hepatocellular carcinoma were analyzed. The characteristics of the patients and the status of the patient at the time of evaluation are shown in Tables 1 and 2.

**Table 1:** Characteristics of patients, total (*n* = 56)

Variables	
Mean age in years (mean ± SD)	60.84 ± 13.12
Age range (years)	19–87
Gender, (*n*, %)	
Male	50 (89.2%)
Female	6 (10.7%)
Underlying liver diseases, *n* (%)	
Chronic liver disease	4 (7.1%)
Cirrhotic	19 (33.9%)
HBS Ag-positive	6 (10.7%)
Unknown	27 (48.2%)

*SD*-Standard deviation

**Table 2:** Status of patients at time of FDG PET CT, *n* (%), total = 56

Initial evaluation	30 (53.6%)
Post-local treatment (surgery/RFA/TACE)	16 (28.6%)
On systemic therapy	5 (8.9%)
Post-liver transplant	5 (8.9%)

*FDG PET CT*-fluoro deoxy glucose positron emission tomography, *RFA*-radiofrequency ablation, *TACE*-transcatheter arterial chemoembolization

### ^18^F FDG PET CT protocol

All patients had undergone a standard protocol for whole-body FDG PET CT following an informed consent, overnight fasting, and with serum glucose levels below 150 mg/dl at the time of injection of FDG. All patients were screened for their renal status prior to using contrast for diagnostic CT part of PET study. ^18^F FDG PET CT images from head to mid-thigh had been acquired on GE Discovery PET 8 slice CT scanner an hour later following intravenous injection of 8–10 mCi of FDG. A breath hold high-resolution non-contrast CT chest was also acquired. Diagnostic CT images were acquired craniocaudally with a linear speed of 27 mm/rotation and a slice thickness of 3.7 mm. The peak voltage was 120 kV, effective variable current strength was between 250 and 350 mA and the pitch was 1.35:1. Intravenous contrast was administered approximately 1.5 ml/kg in volume, administered at a rate of 1.3 ml/s with a scan delay of 35–45 s. Subsequently, PET images were acquired (approx. 8-bed positions of 15 cm length, each of 3 min duration) in caudocranial direction. The data sets were reconstructed using iterative reconstruction technique and co-registered images were displayed on ADWPET workstation

### Image interpretation

Both FDG PET and CT data sets were evaluated by two experienced nuclear medicine physicians and one radiologist in consensus, by visual inspection of the images as well as by semi-quantitative analysis (standardized uptake values, SUVmax in gm/ml). Prior and follow-up imaging findings, relevant surgical and pathology records as and when available were reviewed and correlated. Biopsy or cytological confirmation, detection of new lesions not seen on prior imaging, disease progression, interval size increase, or clinical correlation with or without increased AFP were taken as confirmation of metastatic involvement of the findings suggestive of extrahepatic metastases on FDG PET CT (Table 3).

**Table 3:** Corroboration of lesion(s) seen on FDG PET CT, *n* (%)

Clinical context	15 (26.8)
Cytology	2 (3.6)
Detection of new lesion(s)	8 (14.3)
High AFP and clinical context	15 (26.8)
Histopathology	8 (14.3)
Interval increase in size of lesion(s)	2 (3.6)
Progression on follow-up imaging	6 (10.7)
Total number of patients	56 (100.0)

*AFP*-Alfa fetoprotein

### Data analysis

Distribution of sites of metastatic involvement (organs/lymph nodes), locations, sizes if applicable, CT characteristics, FDG avidity and standardized uptake value of FDG (SUV in gram per milliliter values) of the extrahepatic metastases wherever applicable were documented. Involved regional lymph nodes, distant lymph nodes, and all other sites of distant organ metastatic involvement were classified according to location. Multiple sites of metastatic involvement in an organ or in bilateral organs were considered as a single site.

## Results

The most frequent site of metastatic involvement was lungs, seen in 55.3% of patients. Second most common involvement was regional lymph nodes, seen in 44.65% of patients. Third most common involvement was in distal lymph nodes seen in 39.2% of patients. Skeletal involvement was seen in 32.1% of patients (distribution as shown in Table 8). Other sites of metastatic involvement were adrenal glands (12.5%), peritoneum (3.5%), brain (1.79%), and spleen (1.79% of patients) (Table 4).

**Table 4:** Distribution of overall extrahepatic metastases of HCC on FDG PET CT in 56 patients

Site of involvement	FDG-positive lesions	FDG-negative lesions	Both FDG-positive and FDG-negative lesions	Total
	*n* (%)	*n* (%)	*n* (%)	*n*(%)
Lung	4 (7.14%)	14 (25%)	13 (23.21%)	31/56 (55.3%)
Skeletal	17 (30.36%)	1 (1.79%)	0	18/56(32.1%)
Peritoneum	0	2 (3.57%)	0	2/56(3.5%)
Brain	1 (1.79%)	0	0	1/56(1.79%)
Spleen	1 (1.79%)	0	0	1/56 (1.79%)
Adrenal gland	5 (8.93%)	2 (3.57%)	0	7/56(12.5%)
Muscle	1 (1.79%)	0	0	1/56 (1.79%)
Lymphnodal metastases				
Regional lymph nodes	15 (26.79%)	5 (8.93%)	5 (8.93%)	25/56 (44.65%)
Distant lymph nodes	14 (25%)	5 (8.93%)	3 (5.36%)	22/56 (39.2%)

### Regional metastatic lymphadenopathy

44.65% of patients had regional nodal metastases. Nodal site specific distribution of regional metastatic lymph nodes is enumerated in Table 5. Frequently involved regional metastatic lymphadenopathy was seen at aortocaval (25%), paraaortic 23.21%, portocaval (21.4% of patients), and left gastric (17.8%) stations. 10.7% (6/56) patients had conglomerate regional metastatic lymph nodes. Sizes of the regional nodes ranged from subcentimetric, mostly centimetric, and to maximum of 9 cm in highest dimension for conglomerate lymph nodes. 8.92% (5/56) patients had necrotic regional nodes. Of those patients with regional lymph nodes, 26.79% (15/56) of patients had FDG avid lymph nodes. 8.93% (5/56) had FDG non-avid lymph nodes and remaining 8.93% (5/56) patients had both FDG avid and FDG non-avid lymph nodes (Table 4).

**Table 5:** Distribution of overall extrahepatic regional lymphnodal metastases of HCC on FDG PET CT in 56 patients

Lymphnodal Involvement	*n*(%)	Size range (cm)	FDG + ve	FDG−ve	SUVmax range (gm/ml)	Nec	Non-Nec
Periceliac	6(10.7%)	< 1 cm–3.2 cm	3 (5.3%)	3(5.3%)	2–6.1	1 (1.7%)	5 (8.9%)
Periportal	8(14.2%)	1 cm–4.7 cm	5 (8.9%)	3 (5.3%)	6–13.6	0 (0%)	8 (14.2%)
Porta hepatis	5(8.9%)	< 1 cm–3 cm	4 (7.1%)	1(1.7%)	4–7.9	0(0%)	5 (8.9%)
Paraaortic	13(23.21%)	< 1 cm–3.3 cm	10 (17.8%)	3 (5.3%)	2.3–11.2	1(1.7%)	12 (21.4%)
Portocaval	12(21.4%)	< 1 cm–4.7 cm	6 (10.7%)	6 (10.7%)	3.8–13.6	0(0%)	12 (21.4%)
Peripancreatic	4 (7.1%)	1.2 cm–5.7 cm	3 (5.3%)	1 (1.7%)	4.2–10.5	0(0%)	4 (7.1%)
Aortocaval	14(25%)	< 1 cm–5.5 cm	11 (19.6%)	3 (5.3%)	2.4–11.5	0(0%)	14 25.0%)
Retrocaval	4 (7.1 %)	< 1 cm–1.8 cm	3 (5.3%)	1 (1.7%)	6.8–9.6	1(1.7%)	4 (7.1%)*
L eft Gastric	10(17.8%)	< 1 cm–2.5 cm	8 (14.2%)	2 (3.5%)	2.1–6.7	1(1.7%)	9 (16.0%)
Pericaval	3(5.3%)	1.6 cm– 9 cm	2 (3.5%)	1 (1.7%)	4.2–5.6	0(0%)	3 (5.3%)
Retrocrural	3(5.3%)	1.1 cm–2 cm	3 (5.3%)	0 (0%)	4.6–5.3	1 (1.7%)	2 (3.5%)

*FDG*-fluoro deoxy glucose, *SUVmax* standardized uptake value gm/ml, *Nec*-necrotic

*Patient with necrotic nodes also had non-necrotic lymph nodes

### Distant lymphnodal metastases

In 22 out of 56 patients, 39.2% distant lymph nodes were involved. Commonest site of distant lymphnodal involvement were paratracheal 12.5% (7/56), juxtaphrenic 8.9% (5/56), and mesenteric lymph nodes 7.1% (4/56). Other distant nodal site-specific distribution of regional metastatic lymph nodes is as enumerated in Table 6. Sizes of the regional nodes ranged from subcentimetric, mostly centimetric, and to maximum of 4.8 cm in highest dimension for conglomerate lymph nodes. 1.79 % (1/56) patients had conglomerate regional metastatic lymph nodes. Metastatic lymph nodes in remaining patients were discrete. 3.57% (2/56) patients had necrotic distant metastatic lymph nodes. Twenty-five percent (14/56) of patients had FDG avid lymph nodes. 8.93% (5/56) had FDG non-avid lymph nodes and 5.36% (3/56) patients had both FDG avid and FDG non-avid lymph nodes (Table 4).

**Table 6:** Distribution of overall extrahepatic distribution of distant lymphnodal metastases of HCC on FDG PET CT in 56 patients

Lymphnodal involvement	*n*(%)	Size range (cm)	FDG + ve	FDG-ve	SUVmax range (gm/ml)	Necrotic	Non-necrotic
Lower cervical	3(5.3%)	0.9 cm–1.5 cm	3 (5.3%)	0 (0%)	1.8–7.5	0(0%)	3(5.3%)
Subcarinal	3(5.3%)	1.3 cm–4.8 cm	3(5.3%)	0(0%)	2.5–16.8	0(0%)	3(5.3%)
Paratracheal	7(12.5%)	< 1.2 cm–2.5 cm	6 (10.7%)	1 (1.7 %)	2.5–8.6	1(1.7%)	7 (12.5%)*
Hilar	3(5.3%)	1.1 cm–1.7 cm	2 (3.5%)	1(1.7 %)	2.9–8	0(0%)	3(5.3%)
Interlobar	1(1.7%)	0.9 cm	1(1.7%)	0(0%)	3.3	0(0%)	1(1.7%)
Paracardiac	1(1.7%)	0.7 cm	0(0%)	1(1.7 %)	-	0(0%)	1(1.7%)
Juxtaphrenic	5(8.9%)	1.1 cm–2 cm	3(5.3%)	2(3.5 %)	2.1–3.5	0(0%)	5 (8.9%)
Mesenteric	4(7.1%)	< 1 cm–1.9 cm	1(1.7%)	3(5.3 %)	5.4	0(0%)	4 (7.1%)
Iliac	1(1.7%)	< 1 cm	1(1.7%)	0(0%)	4.2	0(0%)	1(1.7%)
Interlobar	2(3.5%)	1.1 cm–1.6 cm	2(3.5%)	0(0%)	3.3–4.9	1(1.7%)	1(1.7%)
Common iliac	3(5.3%)	1.5 cm–2 cm	3(5.3%)	0(0%)	6.6–9.5	0(0%)	3(5.3%)

*Patient with necrotic nodes also had non necrotic lymph nodes

### Pulmonary metastases

Table 7 shows summary of distribution, pattern of involvement, and imaging features of pulmonary metastatic nodules in 31 patients detected with metastatic pulmonary nodules amongst the 56 patients. In 53.5% (30/56) patients, metastatic nodules were well-defined solid lesions. Only one patient had ground glass nodules. Bilateral involvement was seen in 25 out of 56 patients (44.6%). Lower lobes along with other lobes were more frequently involved (in 23/56, 41.07% patients). Centimetric-sized pulmonary nodules between 1 and 3 cm were more frequent, seen in 16/56 (28.5%) patients while 5/56 (8.92%) patients had > 3 cm lesions. Pleural involvement was seen in 4 out of 56 patients (7.14%). While in 14 out of 56 patients, lung nodules did not show FDG uptake, 13 patients had both FDG avid and non-avid pulmonary nodules. SUVmax range among FDG avid nodules was 5.56 ± 3.94 gm/ml. Patients with distant lymphnodal metastases irrespective of regional nodal involvement showed higher pulmonary metastatic involvement 10/56(17.85%) while those without regional or distant nodal metastatic involvement showed the least pulmonary metastatic involvement 1/56 patient(1.7%).

**Table 7:** Patterns of pulmonary metastases on FDG PET CT, *n* = 56

**a. Involvement** ***n*** **(%)**	Unilateral	6 (10.71%)
	Bilateral	25 (44.64%)
**b. Location,** ***n*** **(%)**	lower lobes only	5 (8.92%)
	lower lobes plus other lobes	23 (41.07%)
	other than lower lobes	3 (5.35%)
**c. Pleural involvement,** ***n*** **(%)**	Yes	4 (7.14%)
	No	52 (92.85%)
**d. Sizes,** ***n*** **(%)**	< 1 cm	10 (17.85%)
	1–3 cm	16 (28.5%)
	> 3 cm	5 (8.92%)
**e. Morphology**	Solid	30(53.5%)
	Ground glass	1(1.79%)
**e. FDG avidity,** ***n*** **(%)**	Positive	4 (7.14%)
	Negative	14 (25.0%)
	Both FDG-positive and FDG-negative	13 (23.21%)
**f. SUV (gm/ml)**		5.56 ± 3.94
**h. Pattern of presence lung nodules,** ***n*** **(%)**	Pulmonary nodules only	8 (14.28%)
	Pulmonary nodules with regional lymph nodes only	5 (8.92%)
	Pulmonary nodules with distant metastatic lymph nodes with or without regional metastatic lymph nodes	10 (17.85%)
	Pulmonary nodules with lymphnodal and other distant metastases	7 (12.50%)
	Pulmonary nodules with distant metastases without nodal involvement	1 (1.7%)

### Skeletal metastases

Tables 8 shows the summary of distribution of skeletal metastases and Table 9 shows pattern of involvement and FDG PET CT imaging features of skeletal metastases. Eighteen out of 56 patients (32.1%) were detected with skeletal metastases (Table 4). Common sites of metastatic skeletal lesions are vertebrae (9/56,16.7%), pelvis (8/56,14.2%) and ribs (6/56, 10.7% patients). Less frequently involved sites are sternum (3/56, 5.36% patients), skull, scapula and long bones (2/56, 3.5% patients each). Nine patients had lytic lesions without soft tissue component and seven patients had lytic lesions with associated soft tissue component. Four patients had lesions without any detectable abnormality on CT scan in the corresponding site. Skeletal lesions were FDG-positive in 16 out of 56 patients (28.57%). SUVmax range was 5.35 ± 2.11 gm/ml. Only one skeletal metastatic lesion in one patient was not FDG-positive. Skeletal metastatic lesions without any nodal or other extrahepatic metastatic involvement was seen in 9 out 56 (16.07%) patients. Seven out of fiftysix (12.5%) patients had skeletal metastases along with other distant metastases.

**Table 8:** Distribution of sites of skeletal metastases, *n* (%)*

Skull	2 (3.57%)
Vertebrae	9 (16.07%)
Pelvis	8 (14.29%)
Sternum	3 (5.36%)
Ribs	6 (10.71%)
Scapula	2 (3.57%)
Long bones	2 (3.57%)

*Many patients had more than one skeletal site involvement

**Table 9:** Characteristics of skeletal metastatic lesions, *n* = 56

**a. Lesion appearance on CT**	***n*** **(%)**
Lytic lesion(s) without associated soft tissue	9 (16.07%)
Sclerotic lesion(s)	0
Lytic lesion(s) with associated soft tissue	7 (12.5%)
No CT abnormality	4 (7.14%)
**b. FDG avidity**	***n*** **(%)**
FDG-positive lesion(s) only	16 (28.57%)
FDG-negative lesion(s) only	1 (1.79%)
FDG-positive and FDG-negative lesion(s) both	0
**c. SUVmax (gm/ml)**	5.35 ± 2.11
**d. Association**	***n*** **(%)**
Skeletal metastasis with lymph nodes	1(1.79%)
Skeletal metastasis with other distant metastases	7 (12.50%)
Only skeletal lesions	9 (16.07%)

### Adrenal gland metastases

Adrenal gland metastases were seen in 7 out of 56 patients, (12.5%) (Table 4). Six out of seven patients had unilateral adrenal gland involvement. Bilateral adrenal gland involvement was seen in one patient with no CT abnormality in one adrenal gland which had only a metabolically active lesion. All lesions on CT were larger than 1 cm. Among them three patients had lesions larger than 4 cm. Five lesions in five patients were FDG-positive with a SUV range of 5.74 ± 2.16 gm/ml (Table 10).

**Table 10:** Characteristics of adrenal gland metastases, *n* = 56

		*n* (%)
a. Involvement	Unilateral	6 (10.71%)
	Bilateral	1 (1.78%)
b. CT abnormality	Present	6 (10.1%)
	Absent	1 (1.78%)
c. sizes	< 1 cm	0 (0%)
	1–4 cm	4(7.1%)
	> 4 cm	3 (5.3%)
d. FDG avidity	FDG-positive lesion(s) only	5 (8.92%)
	FDG-negative lesion(s) only	2 (3.57%)
	FDG-positive and FDG-negative lesion(s) both	0 (0%)
e. SUV (gm/ml) range		5.74 ± 2.16

Peritoneal/omental metastases was seen in 2 out of 56 patients and in both patients lesions were FDG non-avid. Lesions were multiple in both patients and largest dimensions were 1.8 cm and 2.5 cm, respectively. Other nodal and distant metastases in both these patients were also FDG-negative.

Brain, spleen, and muscle metastatic lesions were seen in 1 patient each out of 56 patients (1.79%). Brain lesion in left precentral gyrus with enhancement  measured 0.9 cm × 0.9 cm, FDG-positive with SUVmax of 5 gm/ml. Lesions in spleen were multiple, hypodense and FDG positive with SUVmax of 5.3 gm/ml , largest lesion being 1.7 × 1.6 cm in dimensions. A muscle lesion was seen in a patient deep to pectoralis muscles at their sternal attachments on the left side and was 5.8 cm × 2.3 cm in dimensions, enhancing, FDG-positive with SUVmax of 7.4 gm/ml.

Relative pattern of involvement of metastatic lesions across various categories of patients is shown in Table 11.

**Table 11:** Relative pattern of involvement of metastatic lesions across various categories of patients, *n* = 56

Metastatic involvement	Initial evaluation *n* (%)	Post-local treatment (surgery/RFA/TACE) *n* (%)	On systemic therapy *n* (%)	Post-liver transplant *n* (%)
RLN only	3 (5.3%)	–	–	–
RLN and DLN	6 (10.7%)	–	–	–
RLN, DLN, and distant metastases	6 (10.7%)	1(1.7%)	1(1.7 %)	–
RLN and distant metastases (no DLN)	6 (10.7%)	–	1(1.7 %)	1(1.7%)
DLN and distant metastases (no RLN)	3 (5.3%)	5 (8.9%)	–	1(1.7%)
Distant metastases only (no DLN or RLN)	6 (10.7%)	10 (17.8%)	3 (5.3%)	3 (5.3%)

*RLN* regional lymph nodes, *DLN* distant lymph nodes

## Discussion

Our analysis showed that 56 of the 224 patients evaluated in this study showed extrahepatic metastases. Pulmonary metastases, regional lymphnodal, distant lymphnodal, and skeletal metastases are the most frequent sites of metastatic involvement in our population similar to as reported in literature [9]. Peritoneal/omental, splenic, muscle, and brain metastases were also seen, however, in relatively less number of patients.

^18^F FDG PET CT is traditionally not considered the ‘ideal’ tool for evaluation of HCC, and this is reflected in the relatively small number of consecutive patients (in this study) who were referred to us for a FDG PET CT evaluation over a period of nearly 8 years. Newer PET tracers are being investigated and a few of them such as ^18^F choline have become part of clinical practice. However, the synthesis of ^18^F choline is a relatively more expensive affair when compared to the synthesis of ^18^F FDG, and this is a major determinant for its regular availability especially in developing nations. It is due to this reason that most often, FDG still remains as radiopharmaceutical in use for HCC evaluation. It is in this context that this study was undertaken to demonstrate the ability of FDG PET CT in correctly identifying extrahepatic metastases in HCC. And further to use this data to highlight how this cost-effective tool is being grossly underutilized.

Pulmonary metastases were the most common of all extrahepatic metastases of HCC. Involvement of lower lobes of both lungs was the most frequent pattern as usually seen with hemogeneous metastases from extrathoracic primaries [10], possibly due to preferential blood flow induced by gravity [11]. Tumor cells are believed to transit through inferior vena cava or via lymph through main or right thoracic duct to the lungs [12]. A very small percentage of patients also had metastatic nodules in lobes of lungs other than lower lobe. Unilateral lung involvement was also seen (Table 7). Pulmonary parenchymal metastases were predominantly 1–3 cm in size. Pulmonary nodules appeared solid except in one patient where ground glass appearance was also seen. Only in 4 patients out of 56 patients (7.14%) pulmonary nodules were predominantly FDG avid with SUV maximum range of 5.56 ± 3.94 gm/ml which is fairly high enough to convincingly characterize a pulmonary lesion as a metastatic pulmonary nodule. Partial volume averaging effect, signal dilution of small lesions, restricted resolution of PET scanners, effects of respiratory motion, and low tumor cell-to-background ratio are the reasons cited for false-negative FDG uptake in metastatic pulmonary nodules [13]. In all three patients of ours where biopsy confirmed pulmonary metastases, the size of nodules was more than 3.8 cm in largest dimension. FDG positivity in equivocal small pulmonary nodules on CT can be highly suggestive of metastatic involvement as biopsy is not always feasible to confirm either due to small size or difficult locations. Pleural metastatic involvement was also seen in a few patients and is a known entity with a potential to create hemothorax [14].

Regional lymphadenopathy was seen in 44.65% of our patients. Aortocaval, paraaortic, portocaval, and left gastric stations were more commonly seen to be involved. Discrete lymphadenopathy was more common while conglomerate regional metastatic lymph nodes were also seen. Sizes of the regional nodes ranged from subcentimetric, mostly centimetric, and to maximum of 9 cm in highest dimension for conglomerate lymph nodes. Necrotic regional nodes were also seen in a small percentage of patients. Central necrosis has been suggested as a sign of malignant involvement [15]. Regional metastatic lymphadenopathy was seen in majority of our patients. However, it was interesting to see that even in absence of regional lymphnodal metastases,  distant metastases were seen with or without distant metastatic lymphadenopathy (Table 11).

Distant lymphnodal involvement was seen in 39.2% of our patients which included mediastinal and even lower cervical stations. Commonest sites of distant lymphnodal involvement were paratracheal, juxtaphrenic, and mesenteric lymph nodes. Mediastinal metastatic lymphadenopathy is known to be common [15]. Other distant nodal site specific distribution of regional metastatic lymph nodes is as enumerated in Table 6. Sizes of the distant lymph nodes ranged from subcentimetric, mostly centimetric, and to maximum of 4.8 cm in highest dimension for conglomerate lymph nodes. Metastatic distant lymph nodes were predominantly discrete, and only one patient had conglomerate distant metastatic lymph nodes. Necrotic distant metastatic lymph nodes were rare, seen only in two patients. FDG avid distant lymphadenopathy was more common. However, FDG non-avid lymph nodes as well as a combination of both FDG avid and non-avid was also seen (Tables 5 and 6). The fact that distant lymphnodal metastases are predominantly metabolically active and that in HCC distant nodal/extranodal metastases are not uncommon in absence of regional metastatic lymphadenopathy, may suggest a greater role of FDG PET CT in the form of determining biopsy or FNAC site in evaluation of extrahepatic involvement of HCC. In one of our patients, a small supraclavicular lymph node of 1.5 × 1.2 cm size with an SUVmax value of 7.5 gm/ml was proven to be metastatic on FNAC.

A third of patients with HCC and extrahepatic metastasis are known to present with or develop skeletal metastasis over time [16], and they are known to be lytic, hypervascular, and often expansile with lumbosacral and thoracic spine involvement being most common [17]. Eighteen of our patients were detected with skeletal metastases (32.1%) with vertebra, pelvic bones, and sternum being the most common sites followed by ribs, scapula, and long bones (Table 8). Blood-borne tumor emboli rather than lymphatic spread is attributed for skeletal metastases and seen preferentially in bones with red marrow although coexisting skeletal metastases with neighboring nodal involvement is frequent [18]. Of the 18 patients who were detected with skeletal metastases, 8 patients had only skeletal metastases without any lymphnodal or other metastases. It is believed that increased abdominal pressure causes blood to bypass caval systems and reach vertebral plexus veins and thereafter venous and sinusoidal systems of the bones of the spine, shoulder, skull, and even elbows or knees are known to get involved. The remaining patients with skeletal metastases had lymphnodal or nodal plus other sites of involvement. All anatomically evident skeletal lesions were lytic and sometimes were associated with soft tissue component (Table 9). Sclerotic lesions were not seen in any of our patient and are known to be rare [19]. Interestingly, we had four patients in whom there was no corresponding anatomical lesion at the site of abnormally high focal metabolic activity seen on FDG PET. All skeletal lesions except one lesion were highly metabolically active. Our SUVmax range was 5.35 ± 2.11 gm/ml for skeletal lesions which is fairly high enough to convincingly characterize a lesion as metastatic especially when interpreted along with CT features. Our findings of possible isolated skeletal metastases in a small subset of HCC patients and also absence of anatomical lesions at sites of abnormal focal metabolic activity in bones red flags a possibility that conventional locoregional imaging may simply not suffice to detect skeletal metastatic lesions.

Seven patients (12.5%) were detected with adrenal gland metastases. One patient had bilateral adrenal gland involvement, a reported pattern in literature [20]. Three lesions in two patients were histologically proven. There was anatomical abnormality in seven adrenal glands in seven patients, while in the patient who had bilateral involvement, there was no anatomical abnormality in the other adrenal gland at the site of abnormal focal metabolic activity. Both these lesions were histologically proven to be metastases from HCC at different time points. Four lesions with anatomical abnormality were between 1 and 4 cm sizes whereas three lesions were above 4 cm. Two lesions showed mild enhancement on the venous phase CT, while in remaining five patients, there was no enhancement. Five patients (8.9%) had significant abnormal metabolic activity with a SUV range of 5.74 ± 2.16 gm/ml while there was no metabolic abnormality in lesions in two patients. Three lesions larger than 4 cm were all FDG avid while only two of the lesions within 1–4 cm were FDG avid. The smallest lesion which was FDG avid was 2.0 × 1.3 cm. Most of the adrenal gland metastases tended to be metabolically active even in the 1–4 cm range which exemplifies the ability of metabolic imaging to characterize small adrenal lesions.

Peritoneal deposits were detected in only two of our patients (3.5%) although a higher frequency of almost up to 16% have been reported [21]. Deposits were well defined and nodular. They were multiple in number which is a more common pattern although solitary implants are also known [21]. The highest dimensions were 1.8 cm and 2.5 cm, respectively. However, the patients did not have FDG uptake in the lesions. One of the patients had an exophytic component in the primary HCC lesion with regional metastatic lymphadenopathy. Direct invasion from an exophytic tumor and hematogenous transfer through variceal collateral pathways have both been suggested as mechanism of peritoneal dissemination [22]. The other patient was a post-liver transplant recipient and had regional metastatic lymphadenopathy and distant pulmonary, skeletal, and adrenal metastases.

Metastatic involvement of spleen in HCC is known to be rare [23]. We had only one patient with splenic metastasis. Both patterns of single and multiple discrete nodular involvement in spleen are known [18]. In our patient, splenic lesions were multiple, appearing to be hypodense and significant metabolic activity (SUVmax of 5.3 gm/ml) along with a focal metabolically active lesion in right acetabulum. These were new lesions compared to previous FDG PET along with metabolically active recurrence in the post-radiofrequency ablation (RFA) site and alpha fetoprotein(AFP)level of 10,000 ng/ml. Hematogeneous spread through splenic arterial blood flow, through the splenic vein (in patients with portal hypertension) or even by lymphatics in a retrograde fashion have been suggested [11].

Metastasis to muscles is infrequently seen in chest wall and paraveretebral muscles [18]. In one of our patients, a heterogeneously enhancing lesion measuring 5.8 cm × 2.3 cm was seen deep to the pectoralis muscles at the sternal attachment overlying the costochondal junctions with minimal erosions of the calcification along the anterior margin of 4th costochondral cartilage. There was heterogeneous FDG distribution with SUVmax of 7.4 gm/ml. New focal ill-defined hypodensities in liver were detected in comparison to previous CT and were metabolically active. The patient had presented with an increasing left anterior chest wall swelling within a period of 4 years after radiofrequency ablation of HCC in segment V/VI. Subsequently, USG-guided FNAC from anterior chest wall swelling suggested it to be a poorly differentiated neoplasm, possibly poorly differentiated carcinoma.

Central nervous system metastases are reportedly rare. Reported incidences range from 0.6 to 7.7% with brain parenchymal metastasis being the most common amongst CNS manifestations [9]. In our study, in 1 patient, a solitary enhancing lesion 9 mm × 9 mm in precentral gyrus with perilesional edema was seen post-left hepatectomy 5 years after surgery and was being investigated after presenting with convulsions. The lesion was moderately metabolically active with SUVmax of 5.0 gm/ml. This patient also had another metabolically active peripherally located solid 4.5 × 4.5 cm mass lesion in the medial segment of middle lobe of right lung with SUVmax of 9.5 gm/ml which was subsequently proven to be metastasis from HCC. There was however no local recurrence. Very rarely leptomeningeal seeding have also been reported [9].

In 26 patients who had undergone local/systemic therapy or post-liver transplant (Table 11), irrespective of the regional metastatic lymphnodal involvement status, all had distant extra nodal metastatic involvement. It is interesting to note that in this group, there were no patients with only metastatic nodal involvement without distant organ metastases. Whereas among the other 30 patients evaluated as a part of initial evaluation, there were 9 patients who only had metastatic lymphadenopathy and no distant metastases. In six patients, however, distant metastases were detected without either regional or distant nodal involvement. But there were 15 patients who had distant metastases along with either regional or distant nodal metastases or with both. The analysis of factors leading to the varied patterns of metastatic involvement was beyond the scope of our study.

## Limitations

The limitations of this study include the following: first, this was a restrospective analysis. Second, not all suggested extrahepatic lesions had a histopathological confirmation since  in many cases other criterias like increase in size of lesions and appearance of new lesions were used to categorize a lesion as metastatic. Third, important determinants of extrahepatic metastases like tumor size, intrahepatic tumor stage, and FDG avidity of primary lesion in liver could not be evaluated or correlated as many patients were already post-local treatment (surgery/RFA or TACE) or post-liver transplant. And it is for this same reason that no attempt has been made to analyze the role of FDG PETCT (FDG avidity) in determining the biological aggressiveness of the HCC.

## Conclusions

Lungs, regional and distant lymph nodes and skeleton are the most frequently involved sites of extrahepatic metastatic hepatocellular carcinoma. Adrenal glands, muscles, brain, and peritoneum are also involved but to a lesser extent. While metastatic lymphadenopathy with distant organ involvement is a common pattern, involvement of distant lymph nodes or distant organ metastases without regional nodal involvement is also seen. FDG PET CT as a whole-body imaging modality is useful in detecting unsuspected extrahepatic metastases, but its potential to accurately determine disease status and guide therapy (especially in developing nations) appears to be grossly underutilized.

## Data Availability

The datasets used and/or analyzed during the current study are available from the corresponding author on reasonable request.

## Abbreviations

HCC: Hepatocellular carcinoma
^18^F-FDG: Fluorine-18 fluoro-deoxy-glucose
PET: Positron emission tomography
FNAC: Fine needle aspiration cytology
mg/dl: Milligram per deciliter
CT: Computed tomography
GE: General Electric
mCi: Millicurie
mm: Millimeter
kV: Kilo volt
mA: Milliampere
ml/kg: Milliliter per kilogram
ml/s: Milliliter per second
SUVmax: Maximum standardized value
AFP: Alpha feto protein
cm: Centimeter
USG: Ultrasonography
TACE: Transarterial chemoembolization
